# An Uncommon Cause of Recurrent Dialysis Catheter-Related Bacteremia

**DOI:** 10.7759/cureus.19220

**Published:** 2021-11-02

**Authors:** Ng Chee Yong, Yeon Wenxiang, Chai Siang Chew, Chow Weien, Debajyoti Roy

**Affiliations:** 1 Renal Medicine, Changi General Hospital, Singapore, SGP; 2 Cardiology, Changi General Hospital, Singapore, SGP; 3 Nephrology, Changi General Hospital, Singapore, SGP

**Keywords:** methicillin-resistant staphylococcus aureus bacteremia, trans-esophageal echocardiogram, catheter related sepsis, vegetations, hemodialysis access

## Abstract

A 54-year-old man on maintenance hemodialysis with recurrent catheter-related bloodstream infections due to *Staphylococcus aureus* was admitted. Multiple prior transthoracic echocardiograms failed to reveal any vegetation. Subsequently on transesophageal echocardiography a mass consistent with fibrin sheath vegetations was identified and a follow-up diagnostic computed tomography (CT) venogram confirmed the presence of a fibrin sheath with vegetations.

## Introduction

An arteriovenous fistula (AVF) is the ideal access for patients on hemodialysis. However, tunneled dialysis catheters (TDCs) are inserted in the large majority at the time of dialysis initiation. The common causes of non-adherence to “fistula first” include late nephrology referral, patient ambivalence, or the initiation of dialysis in an unplanned manner due to a medical or surgical emergency [1]. Dialysis catheters are commonly associated with complications like thrombosis, infection, and central vein stenosis. Fibrin sheaths develop in 42-100% of patients with central venous catheters (CVCs). Repeated episodes of catheter-related bloodstream infections (CRBSIs) may be related to fibrin sheath vegetations. Transesophageal echocardiography (TEE) is more sensitive than transthoracic echocardiography (TTE) in the diagnosis of this condition.

## Case presentation

A 54-year-old male with end-stage renal failure due to diabetic kidney disease presented to the emergency room with high-grade fever and pain in his right testes. His other medical conditions of note included ischemic heart disease with a reduced ejection fraction of 25% and atrial fibrillation on long-term warfarin. He was on long-term TDC after multiple failed AVF. In the prior six months, he had four admissions for CRBSIs. The blood cultures grew methicillin-resistant *Staphylococcus aureus* (MRSA) on each occasion. The practice in our dialysis unit consists of TDC removal, adequate treatment with therapeutic levels of intravenous vancomycin for four weeks, and TTE to screen for valvular vegetations if *S. aureus* is identified. TTE during each of these episodes was negative for valvular vegetations. As the bacteremia resolved within two to three days of antibiotics initiation, a TDC was re-inserted after two consecutive peripheral blood cultures were negative for bacterial growth. 

**Figure 1:**
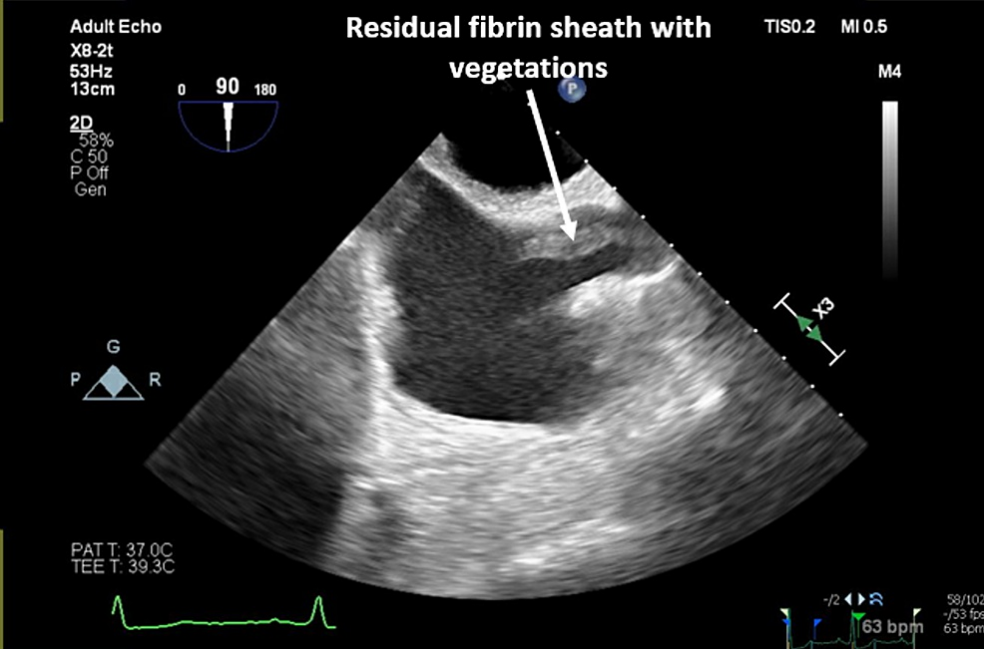
Transesophageal echocardiogram showing fibrin sheath with vegetations (after catheter removal).

**Figure 2:**
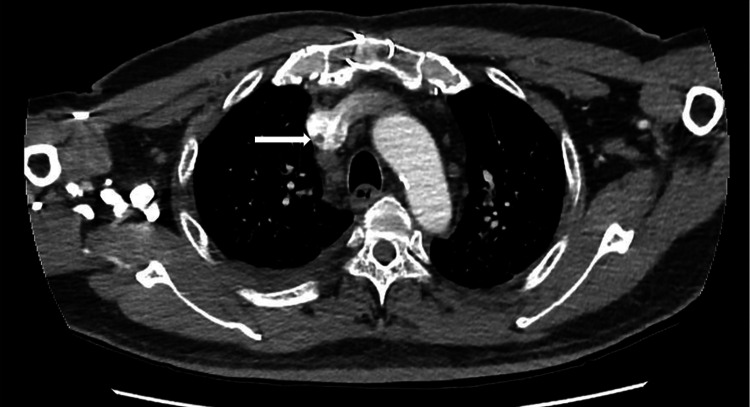
CT venogram demonstrating presence of venous fibrin sheath (after catheter removal). CT, computed tomography.

During his current admission, the blood culture again grew MRSA, and ultrasound of the scrotum was consistent with a small collection in the right testes. As part of the treatment for CRBSI, his right internal jugular TDC was removed and intravenous vancomycin initiated. A repeat TTE on this occasion revealed a large serpentine echo dense mobile mass 2.17 x 0.6 cm seen in the superior vena cava darting in and out of the right atrium consistent with fibrin sheath vegetation. This was confirmed on TEE (Figure 1). A computed tomography (CT) venogram done confirmed the presence of a fibrin sheath (Figure 2). Blood cultures showed clearance at 72 hours after antibiotics initiation and he showed clinical improvement. He was treated with a prolonged six-week course of intravenous vancomycin and is currently contemplating switching to peritoneal dialysis.

## Discussion

TDCs are often unavoidable and up to 80% of patients initiate dialysis using a catheter [2]. Indwelling vascular devices are often associated with the development of a connective tissue sleeve encasing the catheter and often remain intact even after catheter removal. Fibrin sheaths develop in up to 50% of these and can lead to catheter malfunction [3,4]. In an earlier case report, we have described the mechanism of development of a fibrin sheath and have alluded that a calcified sheath can mimic a fractured dialysis catheter [5]. In a case series from New York that reported on 147 patients who underwent thoracic CT scan after removal of CVCs, retained fibrin sheaths were identified in 13.6% of cases of which 45% were calcified [6].

The KDOQI Vascular Access Guidelines 2019 suggests removal of the catheter, antibiotics for four weeks, and TTE if *S. aureus* is grown in the blood culture [7]. Recurrent CRBSI is uncommon but reported causes include catheters with multiple lumens, exchange of catheter over guidewire, presence of fungal infection, and coagulase-negative organism.

Vegetations growing on fibrin sheaths have been reported sporadically [3,4,8]. While CT scan can identify the presence of fibrin sheaths they cannot detect the presence of vegetations associated with infection of endovascular structures of the heart or endocarditis. Similarly, routine TTE is not often useful. While the remnants of fibrin sheath were detected on repeat TTE because of high suspicion for endocarditis and targeted scan in our case and subsequently better delineated on TEE, this finding is often missed. A series from Los Angeles highlighted the challenges of detecting fibrin sheath vegetations in his series of 11 patients where TTE detected none whilst all were visualized on TEE. They further reported a mortality of 36% with death in the following 44-251 days after diagnosis which further reiterates the importance of making the diagnosis [4].* *The present case with recurrent bacteremia in the setting of prior indwelling vascular catheter highlights the importance of TEE in such cases when the TTE is negative. The role of fibrin sheath disruption in such a situation is unclear.

## Conclusions

This case highlights the fact that recurrent CRBSI with the same organism in the setting of prior indwelling dialysis catheter must heighten the suspicion of fibrin sheath vegetation. TEE although more invasive is more sensitive than TTE and should be incorporated into the work-up.
